# Blockchain Applications in the Biomedical Domain: A Scoping Review

**DOI:** 10.1016/j.csbj.2019.01.010

**Published:** 2019-02-08

**Authors:** George Drosatos, Eleni Kaldoudi

**Affiliations:** School of Medicine, Democritus University of Thrace, Dragana, Alexandroupoli 68100, Greece

**Keywords:** Blockchain applications, Biomedical domain, Scoping review, PRISMA-ScR

## Abstract

Blockchain is a distributed, immutable ledger technology introduced as the enabling mechanism to support cryptocurrencies. Blockchain solutions are currently being proposed to address diverse problems in different domains. This paper presents a scoping review of the scientific literature to map the current research area of blockchain applications in the biomedical domain. The goal is to identify biomedical problems treated with blockchain technology, the level of maturity of respective approaches, types of biomedical data considered, blockchain features and functionalities exploited and blockchain technology frameworks used. The study follows the PRISMA-ScR methodology. Literature search was conducted on August 2018 and the systematic selection process identified 47 research articles for detailed study. Our findings show that the field is still in its infancy, with the majority of studies in the conceptual or architectural design phase; only one study reports real world demonstration and evaluation. Research is greatly focused on integration, integrity and access control of health records and related patient data. However, other diverse and interesting applications are emerging, addressing medical research, clinical trials, medicines supply chain, and medical insurance.

## Graphical Abstract

**Figure:**
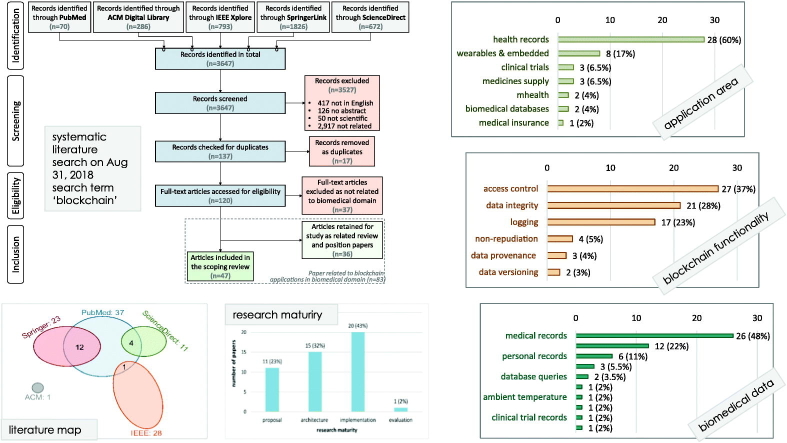

## 1 Introduction

Blockchain technology is on a continuous upward growth trajectory and promises applications in every aspect of information and communications technology [1]. It first appeared as part of Bitcoin cryptocurrency in 2008 [2]; currently, there are more than 2000 cryptocurrencies, more than half of them with a market capitalization more than of 1 million US dollars (based on the Coin Market Cap website for tracking capitalization of cryptocurrencies, https://coinmarketcap.com as accessed on 15 Nov 2018).

**Fig. 1:**
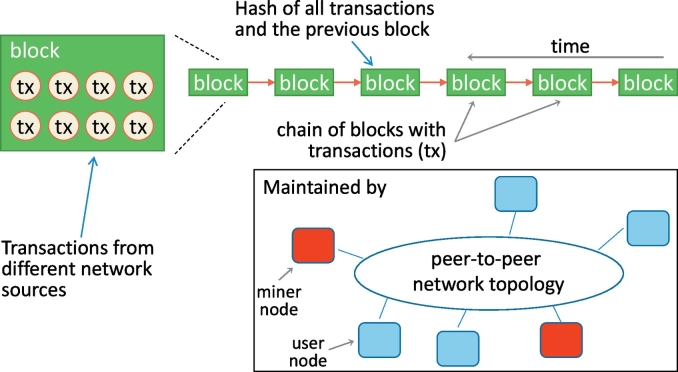
Overview of a blockchain.

The blockchain is defined as a chain of blocks that are time-stamped and connected using cryptographic hashes. A block may contain transactions of many users and generally is publicly available to all users of the network. Additionally, each block contains the hash of the previous block and the transaction data, thus creating a secure and immutable, append-only chain. This chain continuously increases in length as each new block is being added in its end. The blockchain is organized in a peer-to-peer network (Fig. 1) that consists of nodes and each participating node maintains an entire copy of it. An overview of a blockchain is shown in Fig. 1. These nodes can be simple users that want to perform a transaction or validators, called “miners”, that are responsible to verify whether the transactions are valid. The process of agreeing on the contents of the blocks in the chain is referred to as consensus. There are different approaches to reach consensus, a notable example being the Proof-of-Work protocol firstly introduced in Bitcoin. Thorough overviews of blockchain technologies, including blockchain architectures and critical appraisals of consensus algorithms are available in the literature, e.g. [1,[3], [4], [5]].

Based on the access and managing permissions, there are three types of blockchains: public, private and consortium blockchain. A public (or permissionless) blockchain is highly distributed and anyone can participate implementing a miner; this ensures maximum immutability, although limits efficiency due to consensus achieved collaboratively via the highly extended miner network. On the other end, in a private blockchain blocks are processed by miners of a single organization; immutability can be tampered with, but efficiency is maximized. A consortium (or federated) blockchain can provide the efficiency of a private one, while it combines a partially distributed miner network which includes nodes provided by selected organizations.

A large number of blockchain technology frameworks exist (a list of more than 100 is currently available on the Bitcoin Wiki, https://en.bitcoinwiki.org/wiki/Blockchain_Projects_List, accessed on November 19, 2018). Blockchain infrastructures charge for each transaction a fee (known as ‘gas’) proportional to the computational burden that the execution will impose on the blockchain.

A recent critical review of blockchain applications identified the following major application areas [6]: financial services, healthcare, business and industry, digital content distribution, rights management, wireless networks and internet of things security. An overview of blockchain potential and example applications in health are presented in recent relevant reviews [[7], [8], [9]]; these include patient information security, patient data access control, health supply chain management, medical insurance, security in health related internet of things applications and medical education.

In this paper, we systematically analyze the state of the art in blockchain applications in the biomedical domain, as presented in the peer-reviewed scientific literature.

## 2 Methods

### 2.1 Goal and Research Questions

The goal of this systematic literature review is to map the current research area of blockchain technologies as applied to the biomedical domain and identify main sources and types of evidence, their variety and maturity. In particular, this study aims to address the following primary research questions:

1. What areas have been addressed in current applications of blockchain technology in the biomedical domain?
2. What is the level of project maturity in blockchain applications in the biomedical domain?
3. What types of biomedical data have been considered in blockchain biomedical applications?
4. What are the major reasons for using blockchain technology in the biomedical domain?
5. Which blockchain technology frameworks are used for biomedical applications?

### 2.2 Research Protocol

This study follows the scoping review methodology, which, by its definition, is the most suitable knowledge synthesis approach for systematically mapping concepts underpinning a research area and identifying the main sources and types of available evidence [10,11]. The scoping review protocol of this study was drafted using the Preferred Reporting Items of Systematic Reviews and Meta-Analysis (PRISMA) methodology and its extensions for scoping reviews (PRISMA-ScR) [12]. A summary of the protocol is presented in the following subsections.

### 2.3 Eligibility Criteria

To be included in the review, papers needed to report on some aspect of blockchain technology as applied to a biomedical domain problem. Papers were included if they were published in peer-reviewed journals or peer-reviewed conference proceedings written in English and only if they were reporting original research related to biological and healthcare area, irrespective of the maturity level of each published work. Papers were excluded if they did not fit into the conceptual framework of the study; in particular if they were reviews or position papers, or if they reported on blockchain technology applied to support an aspect of a biomedical system/application not directly related to health or biology.

### 2.4 Information Sources and Search Strategy

To identify potentially relevant publications, the following online bibliographic databases were searched on August 31st, 2018: *PubMed* [13], *ACM Digital Library* [14], *IEEE Xplore* [15], *SpringerLink* [16] and *ScienceDirect* [17]. Each database was searched via their proprietary search engine interface using the single keyword “blockchain”. Results were retrieved using the provided export function of each database in CSV format (for PubMed, ACM Digital Library, and SpringerLink) and in BibTeX format (for IEEE Xplore, and ScienceDirect); BibTeX was transformed into CSV using the open source bibliography reference manager *JadRef* [18], and citation details for all papers retrieved were eventually compiled into a single Microsoft Excel file (available upon request).

### 2.5 Selection of Sources

The authors of this paper screened independently the title and abstract of all publications and excluded publications with no title, no abstract, not in English; records not corresponding to publications (e.g. interviews, commentaries, call for special issue papers, editorials, etc.); publications not related to blockchain and publications not related to biomedical domain. When we were not able to discern the above information from the title or abstract, the paper was included for further study. The reviewers discussed their findings and agreed on a consolidated publication list.

Subsequently, the two reviewers studied independently and in detail the full text of all the publications in the list retained after the first screening, in order to agree on a final list of papers related to blockchain technologies in biomedical domain. This final list was studied to identify and organize papers into three pools: (1) research papers; (2) reviews of any type; and (3) position papers. Papers in the first pool were included for this scoping review and further analyzed using the data charting approach presented in the following subsection. Papers in the second and third pool were retained for statistical analysis and further general reference.

### 2.6 Data Charting

A data charting form was developed jointly by the authors to determine which variables to extract. Subsequently, they independently charted the data and discussed the results. Minor discrepancies were resolved by discussion and a consolidated data chart was constructed (available upon request).

For each paper included in the list after the first screening, the following data items were extracted:

- *Year of publication:* as this is stated in the citation exported by the database.
- *Source type:* publication types considered include (a) journal paper; (b) conference proceedings paper; (c) magazine article; and (d) book chapter.
- *Article type:* (a) research papers reporting novel applications of blockchain technologies in the biomedical domain; (b) reviews of any type (narrative, scoping, systematic, etc.); and (c) position papers discussing general aspects of the field, but not reporting on novel research or systematically reviewing existing research.

For each research paper included in the scoping review, further data items where extracted in order to categorize the paper. Since we have not managed to identify another systematic or scoping review on the same topic and research questions, we opted for a topic-specific alternative for the classification of papers, as described in guidelines for systematic mapping studies in software engineering [19]. The authors studied the papers to extract mapping keywords related to the scoping review questions. Through an iterative process, a number of data items were identified and used to construct a classification scheme. The papers were sorted in the identified categories. In the process, data items representing categories were merged or renamed where needed, to refine the ad-hoc classification scheme based on the pool of papers included in the scoping review. Finally, the following additional data items were extracted:

- *Application area:* the specific biomedical area considered in the publication, e.g. health records, clinical trials, medicines, medical evidence databases, medical education.
- *Maturity of approach:* using the following scale (a) research proposal of a novel blockchain application; (b) architectural design of a system or system component employing blockchain technologies; (c) implementation of a working prototype of the proposed blockchain system component, with details on the technical platforms and tools used; and (d) evaluation in the real setting.
- *Biomedical data:* the type of data considered in the proposed blockchain application, e.g. *medical* health records, personal health records, consent forms, drug information, environmental data, location, medical evidence data, etc.
- *Reasons for using blockchain:* to what end blockchain technology is exploited in each application, for example, access control, non-repudiation, data auditing, data provenance, data versioning and integrity.
- *Blockchain technology:* the specific blockchain infrastructure (if any) used or proposed for the implementation, e.g. Bitcoin, Ethereum, Hyperledger Fabric, etc.

### 2.7 Synthesis of Results

We analyzed the overall results after the first screening to present an overview of existing literature in blockchain applications in the biomedical domain. Subsequently, we focused on literature presenting original research in order to identify the breath (application areas, reasons for using blockchain, data types) and depth (maturity level) of existing research. The individual characteristics of each included publication are presented in tabular form. We have also computed and graphed summaries of charted data frequencies. Finally, we summarize and discuss scoping review finding for each of the identified application areas.

## 3 Results

### 3.1 Selection of Sources

**Fig. 2:**
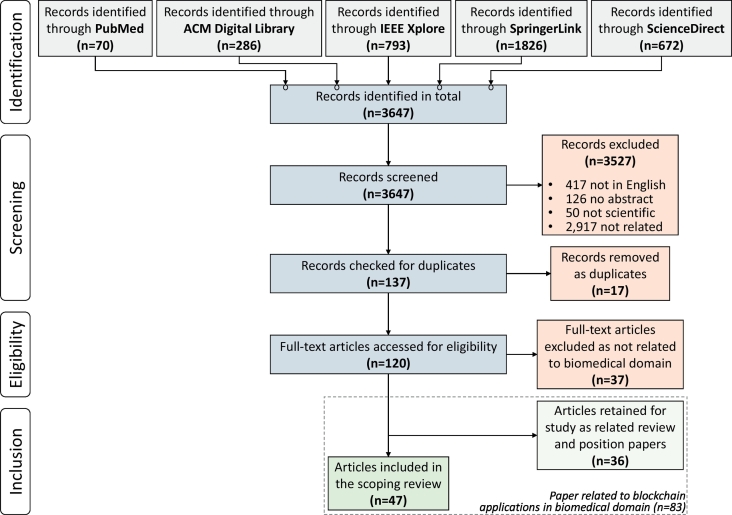
Source selection process from literature databases.

A total of 3647 abstracts were retrieved (70 from PubMed; 286 from the ACM Digital Library; 793 from IEEE Xplore; 1826 from SpringerLink; and 672 from ScienceDirect). After the first screening 3527 papers were excluded: 417 were not in English, 126 had no abstract, 50 were not scientific papers, 2917 were about blockchain technologies but not related to biomedical domain or they were not about blockchain technology at all. Thus, after the initial title and abstract screening, we retained 137 papers for further study. After removing 17 duplicates, 120 unique papers were identified for full paper analysis. During the second screening, 37 papers were further excluded as not relevant to blockchain applications in biomedical domain. From the remaining 83 papers, 5 papers were identified as reviews, 31 papers were identified as position papers, and 47 as research papers. The 47 research papers were included in the scoping review, while the reviews and position papers were retained for further study as papers related to blockchain applications in the biomedical domain. The source selection process is shown in Fig. 2.

**Fig. 3:**
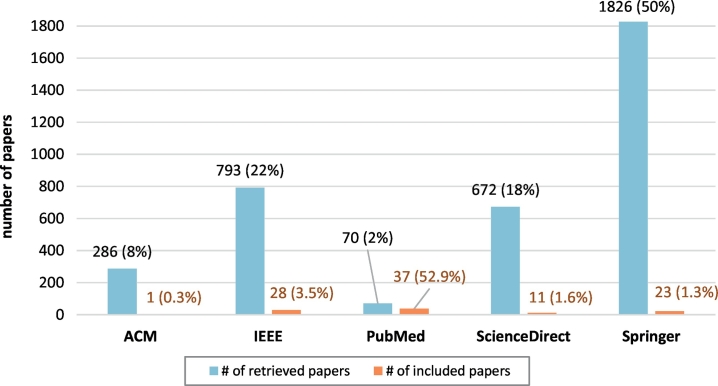
Contribution of the individual bibliographic databases in the pool of papers. For each database, the bar on the left (blue) shows retrieved papers as an absolute number and as the percentage of the total number of retrieved papers. The bar on the right (orange) shows the relevant papers (i.e. papers included and retained) as an absolute number and as the percentage of the total number of papers retrieved from this database. (For interpretation of the references to colour in this figure legend, the reader is referred to the web version of this article.)

**Fig. 4:**
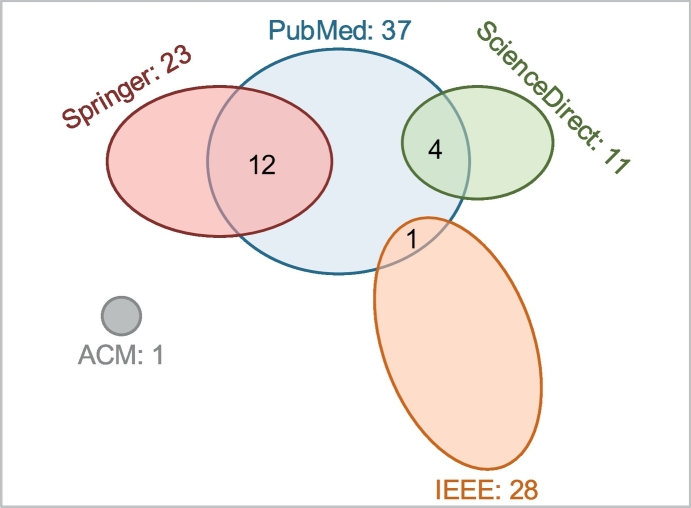
Duplicates among different databases when considering the pool of relevant papers to the topic of blockchain applications in the biomedical domain.

Overall, 3% of the retrieved papers were found relevant to the topic of this study (either included in the scoping review or retained for further study as related to blockchain applications in the biomedical domain). Fig. 3 shows the contribution of each database in the overall pool of papers. SpringerLink returned overall the highest number of papers, corresponding to the 50% of all retrieved papers. The lowest number of retrieved papers corresponds to the PubMed database (2% of all retrieved papers). However, after excluding all irrelevant papers (apart from duplicates), PubMed shows the highest contribution in the pool of relevant papers (46% of all relevant papers), with nearly 53% of the papers retrieved from PubMed being relevant to applications of blockchain in the biomedical domain. When considering the 17 duplicates (after exclusion of irrelevant papers), all databases except for PubMed returned unique results, the overlapping occurring only among PubMed and any other database. Fig. 4 shows the duplicates among the 5 databases in the final pool of papers relevant to the topic of blockchain applications in the biomedical domain.

**Fig. 5:**
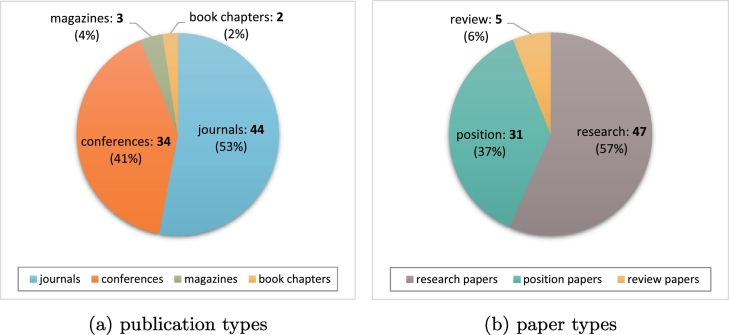
Distribution of papers relevant to blockchain applications in the biomedical domain. The pie chart on the left shows number of papers from different types of publication; the pie chart on the right shows the different types of papers in the collection as tagged after first round of data charting.

Further analysis of the 83 papers related to blockchain applications in the biomedical domain shows that more than half (53%) are journal papers and around 41% are full papers in conference proceedings (Fig. 5a, left chart). Journal papers are scattered in 29 different journals; only 4 journal titles have published more than one paper on biomedical applications of blockchain, namely Journal of Medical Systems (10 papers), Computational and Structural Biotechnology Journal (4 papers), IEEE Access (3 papers) and F1000 Research (2 papers).

**Fig. 6:**
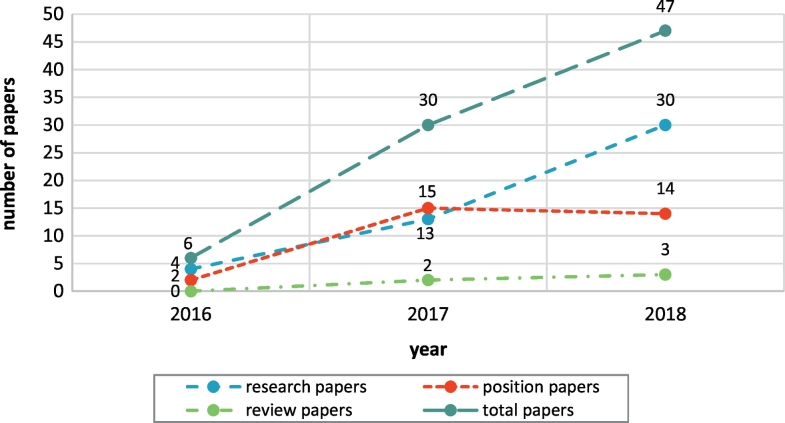
Yearly distribution of the papers relevant to blockchain applications in the biomedical domain, for the different types of papers (review, position, and research). Note that papers were retrieved on August 31, 2018, so data for 2018 are only partial.

The first round of data charting of these papers revealed that more than half of the papers (57%) present novel research; 37% are position papers presenting general discussions on the field and only 6% are reviews of different subdomains (Fig. 5b, right chart). All papers have been published since year 2016 and onwards: 6 (7%) papers published in 2016; 30 (36%) papers published in 2017; and 47 (57%) papers published in 2018. Fig. 6 shows the yearly distribution of the different types of published papers.

### 3.2 Characteristics of Sources and Synthesis of Results

The characteristics and data charted for each of the 47 research papers included in the scoping review are presented in Table 1.

**Table 1:** Research papers included in the scoping review and their characteristics.

Author	Year	Source type	Application area	Maturity
Al Omar A. [20]	2017	Conference	Health records	Proposal
LightGray Angeletti F. [21]	2017	Conference	Clinical trials	Implementation
Archa [22]	2018	Conference	Medicines supply	Architecture
LightGray Azaria A. [23]	2016	Conference	Health records	Implementation
Benchoufi M. [24]	2018	Journal	Clinical trials	Implementation
LightGray Bocek T. [25]	2017	Conference	Medicines supply	Evaluation
Brogan J. [26]	2018	Journal	Wearables & embedded	Implementation
LightGray Castaldo L. [27]	2018	Conference	Health records	Architecture
Cichosz S. [28]	2018	Journal	Health records	Proposal
LightGray Cunningham J. [29]	2017	Conference	Health records	Implementation
Dagher G. [30]	2018	Journal	Health records	Architecture
LightGray Dey T. [31]	2017	Conference	Wearables & embedded	Proposal
Dubovitskaya A. [32]	2017	Conference	Health records	Implementation
LightGray Dubovitskaya A. [33]	2017	Conference	Health records	Implementation
Fan K. [34]	2018	Journal	Health records	Implementation
LightGray Griggs K. [35]	2018	Journal	Wearables & embedded	Architecture
Hussein A. [36]	2018	Journal	Health records	Architecture
LightGray Ichikawa D. [37]	2017	Journal	Mhealth	Implementation
Ji Y. [38]	2018	Journal	Mhealth	Architecture
LightGray Jiang S. [39]	2018	Conference	Health records	Implementation
Juneja A. [40]	2018	Conference	Wearables & embedded	Implementation
LightGray Kaur H. [41]	2018	Journal	Health records	Proposal
Kleinaki A. [42]	2018	Journal	Biomedical databases	Implementation
LightGray Li H. [43]	2018	Journal	Health records	Implementation
Liang X. [44]	2017	Conference	Wearables & embedded	Implementation
LightGray Liang X. [45]	2018	Conference	Health records	Architecture
Liu W. [46]	2017	Conference	Health records	Proposal
LightGray Mangesius P. [47]	2018	Conference	Health records	Architecture
Mense A. [48]	2018	Conference	Health records	Implementation
LightGray Mytis-Gkometh P. [49]	2018	Conference	Biomedical databases	Implementation
Nugent T. [50]	2016	Journal	Clinical trials	Implementation
LightGray Patel V. [51]	2018	Journal	Health records	Architecture
Roehrs A. [52]	2017	Journal	Health records	Architecture
Saravanan M. [53]	2017	Conference	Wearables & embedded	Implementation
LightGray Staffa M. [54]	2018	Journal	Health records	Architecture
Tseng J. [55]	2018	Journal	Medicines supply	Proposal
LightGray Tyndall T. [56]	2018	Conference	Health records	Implementation
Uddin M. [57]	2018	Journal	Wearables & embedded	Architecture
LightGray Wang H. [58]	2018	Journal	Health records	Architecture
Wang S. [59]	2018	Journal	Health records	Proposal
LightGray Xia Q. [60]	2017	Journal	Health records	Proposal
Yue X. [61]	2016	Journal	Health records	Proposal
LightGray Zhang A. [62]	2018	Journal	Health records	Implementation
Zhang J. [63]	2016	Journal	Wearables & embedded	Architecture
LightGray Zhang P. [64]	2018	Journal	Health records	Implementation
Zhang X. [65]	2018	Conference	Health records	Proposal
LightGray Zhou L. [66]	2018	Journal	Medical insurance	Implementation

Research papers included in the scoping review were published either in journals (55%) or in conference proceedings. Journal papers were published in 13 different journals; only 4 journal titles have published more than one paper on biomedical applications of blockchain, namely Journal of Medical Systems (9 papers), Computational and Structural Biotechnology Journal (3 papers), IEEE Access (3 papers) and F1000 Research (2 papers). Conference papers were published in 19 different conference proceedings; only one conference proceedings title published more than one paper included in this scoping review, namely Studies in Health Technology and Informatics.

**Fig. 7:**
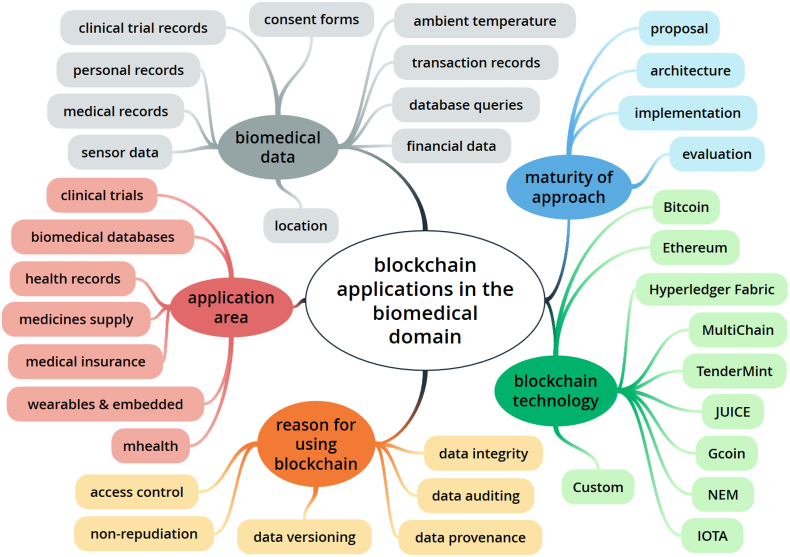
The classification scheme that emerged from the analysis of papers included in this scoping review presented as a mind map.

Based on the iterative identification of data charting keywords (as presented in Section 2.6), a classification scheme emerged from the papers included in the scoping review and is shown in Fig. 7. This figure was created using the online concept mapping tool GoConqr (https://www.goconqr.com).

**Fig. 8:**
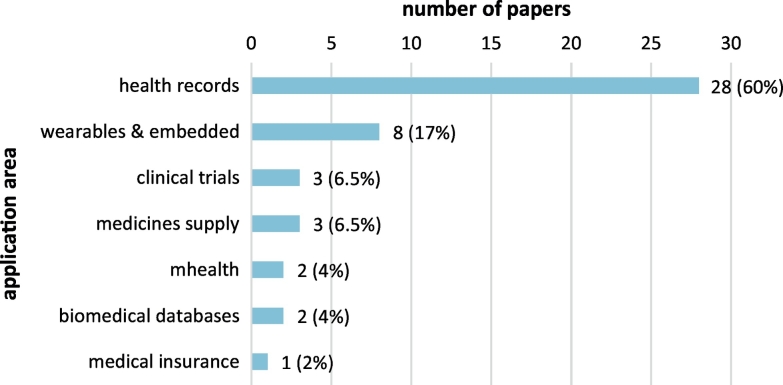
Research areas addressed in the papers included in the scoping review.

**Fig. 9:**
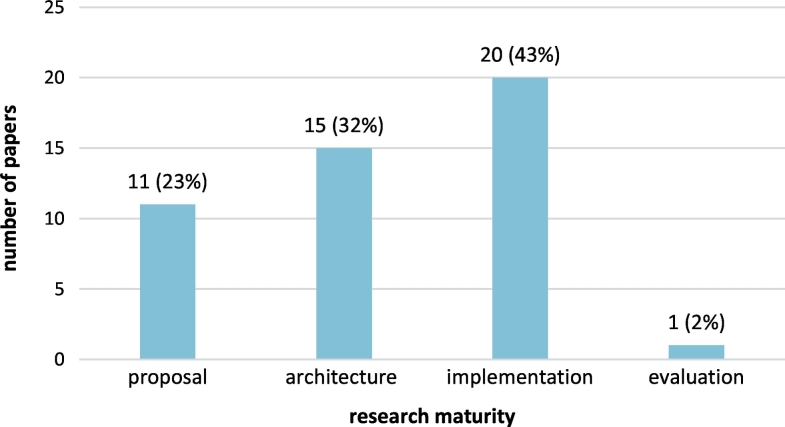
Maturity of the research presented in the papers included in the scoping review.

Overall, published research covers seven distinct application areas in the biomedical domain, as shown in Fig. 8. More than half (60%) of the papers address the application of blockchain technology in health records, including personal and medical health records and their segments. Next favorite application considers wearable and embedded biomedical sensors (17%). Other application areas that are addressed include clinical trials, medicines supply chain, mobile health (mhealth), biomedical databases, and medical insurance. The level of maturity of the research presented in the papers of the scoping review is presented in Fig. 9. Most research is at the implementation (43%) or architecture (32%) phase, while a considerable number of papers (23%) only draft the proposed idea; only one paper presents evaluation of an applied and pilot demonstrated blockchain solution in the biomedical domain.

Analysis of each source identified the specific data considered for the blockchain application, reasons for using blockchain, and the blockchain technology framework (if any) used; a summary of data charted is shown in Table 2.

**Table 2:** Descriptive data on the particular blockchain application presented in each of the papers included in the scoping review; the table presents the type of biomedical data considered in each application, the reason for using blockchain and the blockchain technology framework (in any) considered for the implementation.

Author	Data	Reason for using blockchain	Technology
Al Omar A. [20]	Medical records	Data integrity	N/A
Angeletti F. [21]	Sensor data	Data integrity, access control	Ethereum
Archa [22]	Transaction records	Logging, data provenance	TenderMint
Azaria A. [23]	Medical records	LOGGING, access control	Ethereum
Benchoufi M. [24]	Consent forms	Non-repudiation, logging, data versioning	Bitcoin
Bocek T. [25]	Ambient temperature	Logging	Ethereum
Brogan J. [26]	Sensor data	Access control, data integrity	IOTA
Castaldo L. [27]	Medical records	Logging	MultiChain
Cichosz S. [28]	Personal records, sensor data, medical records	Access control	NEM
Cunningham J. [29]	Medical records	Access control	Ethereum
Dagher G. [30]	Medical records	Access control, data integrity	Ethereum
Dey T. [31]	Sensor data	Data integrity	N/A
Dubovitskaya A. [32]	Medical records	Access control	Hyperledger Fabric
Dubovitskaya A. [33]	Medical records	Access control	Hyperledger Fabric
Fan K. [34]	Medical records	Access control	N/A
Griggs K. [35]	Sensor data	Logging, data integrity	Ethereum
Hussein A. [36]	Medical records	Access control	N/A
Ichikawa D. [37]	Personal records, sensor data	Data integrity	Hyperledger Fabric
Ji Y. [38]	Location	Data integrity	N/A
Jiang S. [39]	Medical records,	Access control, non-repudiation,	Custom
	Personal records	Data integrity	
Juneja A. [40]	Sensor data	Access control	Hyperledger Fabric
Kaur H. [41]	Medical records	Logging	N/A
Kleinaki A. [42]	Database queries	Non-repudiation, data integrity, data versioning	Ethereum
Li H. [43]	Medical records	Data integrity	Ethereum
Liang X. [44]	Sensor data	Data integrity, access control, logging	Hyperledger Fabric
Liang X. [45]	Personal records, sensor data	Access control, data integrity	N/A
Liu W. [46]	Medical records	Data integrity, logging	N/A
Mangesius P. [47]	Medical records	Access control	N/A
Mense A. [48]	Personal records	Access control	Ethereum
Mytis-Gkometh P. [49]	Database queries	Non-repudiation, data integrity	Ethereum
Nugent T. [50]	Clinical trial records, medical records	Data integrity, logging	Ethereum
Patel V. [51]	Medical records	Access control, logging	N/A
Roehrs A. [52]	Personal records	Logging, access control	N/A
Saravanan M. [53]	Sensor data	Access control	Ethereum
Staffa M. [54]	Medical records	Logging	N/A
Tseng J. [55]	Transaction records	Logging, data provenance	Gcoin
Tyndall T. [56]	Medical records	Data provenance	N/A
Uddin M. [57]	Sensor data	Access control, data integrity	Custom
Wang H. [58]	Medical records	Data integrity, logging	N/A
Wang S. [59]	Medical records	Data integrity, access control	N/A
Xia Q. [60]	Medical records	Access control, logging	N/A
Yue X. [61]	Medical records	Access control	N/A
Zhang A. [62]	Medical records	Access control	JUICE
Zhang J. [63]	Sensor data	Access control	N/A
Zhang P. [64]	Medical records	Access control, data integrity	Ethereum
Zhang X. [65]	Medical records	Access control	N/A
Zhou L. [66]	Financial data, transaction records	Data integrity, logging	Ethereum

**Fig. 10:**
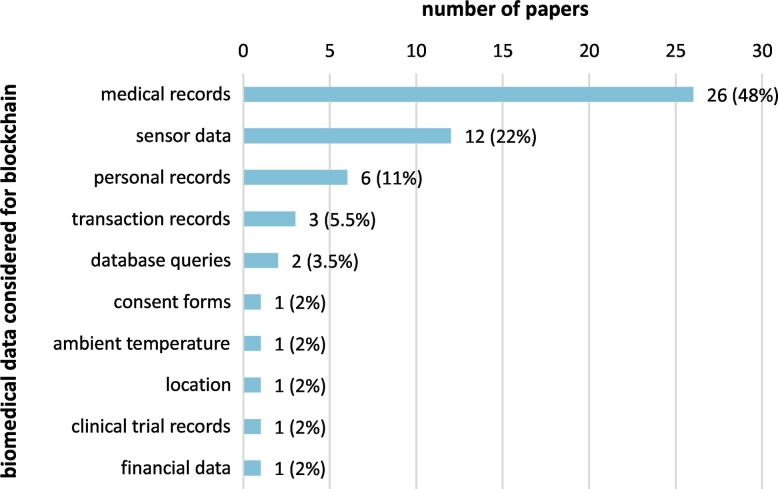
Different biomedical data types considered in the blockchain applications presented in the papers included in the scoping review.

The most prevalent biomedical data type (81%) refers to medical data related to health records, including medical records (48%), personal biosensor data (22%), and personal health records (6%). The rest of proposed solutions consider diverse biomedical data types: clinical trial data (4%), biomedical database queries (4%), ambient measurements such as location and temperature (4%), and financial data (2%). A graph of frequencies for the different biomedical data types considered in the blockchain applications included in the scoping review is presented in Fig. 10.

**Fig. 11:**
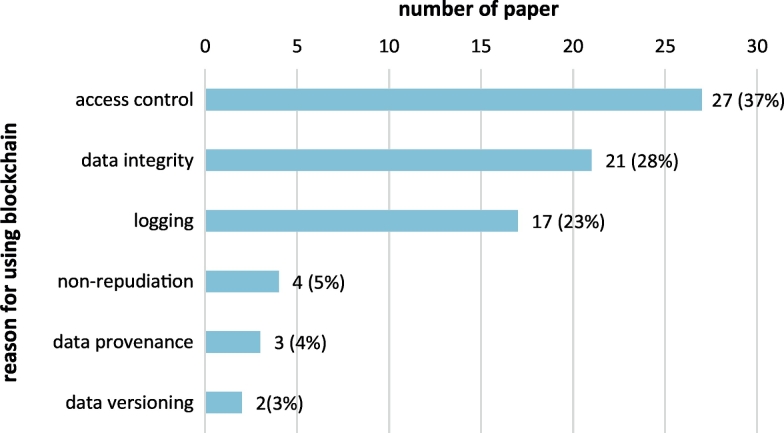
Blockchain functionalities exploited in the papers included in the scoping review.

Blockchain is employed to address several information security components (Fig. 11). Most papers propose blockchain for distributed access control (37%), data integrity (28%), and data and event logging (23%); other uses include ensuring non-repudiation of medical acts or transactions (5%), tracking data provenance (4%) and versioning (3%).

**Fig. 12:**
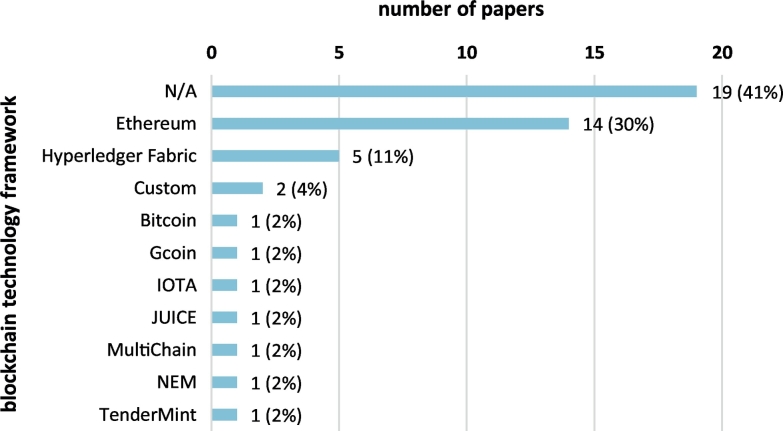
Blockchain technology frameworks considered for the implementation of the papers included in the scoping review.

Fig. 12 shows a graph of frequencies for the various blockchain technology frameworks considered for the implementation of the proposed solutions. The majority of papers (41%) do not report the use of a particular blockchain technology framework; most of these papers are at the proposal or architecture phase. Ethereum [67] is the most commonly used technology framework (30% of scoping review papers), and Hyperledger Fabric [68] is the second common (11%). Other distributed ledger technology frameworks used include Bitcoin [2], Gcoin [69], IOTA [70], JUICE [71], MultiChain [72], NEM [73], and TenderMint [74].

## 4 Discussion

Although several publications have presented an overview of blockchain applications in the biomedical domain, to the best of our knowledge this is the first systematic study covering a large part of the published literature in the biomedical domain. In particular, an earlier review of blockchain applications in 2017 identified some applications related to electronic and personal health care records [6]; the same year, a review on digital solutions to combat fake medicines trade [75] identified blockchain as an emerging technology with potential, and gave an overview of related blockchain applications in the drug supply industry. Three more reviews highlight the potential of blockchain for healthcare in 2018. The first one discusses the potential of blockchain technology to enable electronic health record integration and support medicines supply chain and health claims [7]. Another paper discusses applications in drug supply chain, electronic health records, clinical trials and health insurance transactions [8]. The third review [9] identifies primarily the potential of blockchain to address several different problems in healthcare and presents examples of application of the technology in clinical trials, electronic health records and expands its arguments to the area of research, teaching and digital payments.

Main findings of the scoping review show that blockchain technology has so far been proposed to address several security issues in a number of different biomedical applications as summarized in the following paragraphs.

As defined by the International Organization for Standardization (ISO), electronic health records include any computer processable repository of information regarding the health status of an individual [76]. Although a number of related terms appear in literature [77,78], we can identify two main categories: medical records, produced mainly by hospital departments and generally focused on medical care; and personal health records, controlled by the patient and generally containing information at least partly entered by the patient. Blockchain technologies show a potential to address several security and integration issues regarding health records [20,59]. Based on the results of this scoping review, blockchain technology has been proposed to create ledgers of patient record segments usually residing in different healthcare providers either for patients to create a virtual map of their medical history [23,34] or for medical record integration with the healthcare enterprise [43,47,51,56,58,60,62,64,65] and for sharing record segments across countries [27]. Blockchain has been also used to empower patients and allow them to control and grant access to their medical record segments, either for continuity of care, second opinion, or medical research [23,29,30,[32], [33], [34],^36^,^51^,^61^,^62^]. Ledgers of medical acts, medical data requests, data accesses and other related transactions have been also envisaged in blockchain to allow for non-repudiation of medical acts and other healthcare related activities [39,41,46,54]. Finally, medical data integrity can be ensured by storing hashes in the blockchain [39,43,58].

Blockchains have been proposed to achieve integration of distributed personal health record segments in a unified personal health record [48,52], often also including data from sensors and medical records [39,45]. Another application of blockchain technology in personal health records allows patients to control access to their personal records [45] and share data with third parties [28]. A private blockchain has also been proposed to store personal health records in order to ensure integrity and availability even for health data generated outside the trusted hospital environment [37]. Finally, medical insurance related health record data can be encrypted and immutably stored in the blockchain for future use by the medical insurance industry [66].

Critical appraisal of all health record related studies shows that main reasons for proposing blockchain technology in medical records relate to addressing long lasting medical record integration problems associated either with integrating record segments under a virtually common ledger (the blockchain) or providing a unified mechanism for controlling access to records or their segments. Another important application related to health records considers data integrity and unified logging of medical acts. However, we should note that very often there is a confusion of what data are put in the blockchain. Thus, in many cases, the blockchain is (mistakenly we believe) proposed to store the entire health record data, rather than used as is designed, i.e. to create a rigorous and tamper proof registry of data and of actions.

Overall, blockchain applications in health records are at a rather initial stage of maturity. Less than half (42%) show some degree of implementation, which is limited to laboratory or simulation testing. No study presents a real-world demonstration or evaluation of the proposed solution. We anticipate that as technology matures and more industrial applications emerge, real world pilot demonstrations will help shape the field and will reveal the most suitable applications of blockchain in medical records. Indeed, in 2016, the Estonian Health and Welfare Information Systems Centre launched a project to create and secure a log file of all medical data processing activities in the national health record system using blockchain technology; the project, probably the first nationwide deployment, and is currently in pilot stages [79]. The same underlying blockchain technology is now being used in other industrial products to power personal care records in the UK [80].

The recent trend of quantified self [81] brought attention to personal biosensors (wearables and embedded) creating an exploding amount of complex personal data streams, usually stored in various third-party sensor provider clouds or personal health records. Their potential to transform health care and global public health has drawn attention to addressing technological challenges such as integration with other health data, integrity, ownership, and access control [82]. Thus, blockchain technology has been proposed for storing summaries [26,31,57] or hashes [44] of sensor data as retrieved from sensor providers' clouds to ensure sensor data integrity, and patient ownership. Other approaches employ blockchains to create ledgers of distributed sensor data segments [35,40,63], and allow the patient to control access to personal sensor data [26,40,44,53,57,63]. Indeed, as personal biosensors are gaining popularity and their data are increasingly used for personalized health decision making, the problem of ensuring sensor data integrity will become of greater importance. Blockchain has the potential to easily address this via storing summaries or hashes of data. Personal sensors are expected to be available (as any other consumer goods) for sharing, renting and re-selling; following emerging examples from other consumer areas [83], blockchain can be used to realize smart, virtual locks and pass the controls to each next sensor user or any other interested party.

A different, emerging field of blockchain applications in the biomedical domain refers to supporting research. One particular type involves clinical trials, where blockchain technology has been exploited in three different paradigms. A first application [21] involves blockchains to preserve participant data privacy and integrity while patient is evaluated for trial inclusion, and release access to data after subscription to trial. A second application ensures non-repudiation and versioning of trial consent forms [24], while a third application considers private blockchains for storing all clinical trial data to guarantee trial protocol compliance and trial data integrity [50]. Another contribution to biomedical research considers safeguarding researcher transactions with reference biomedical databases that hold continually submitted and updated scientific evidence (including clinical registries, pharmaceuticals, metabolomics and other omics, and publications). Blockchain technology has been proposed to provide integrity and non-repudiation for reference database queries and respective results [49], and versioning of time evolving database query results [42]. In all these applications, blockchain is employed as a distributed, tamper proof ledger of research activities on specific data, thus ensuring integrity, immutability and non-repudiation of research course. This type application seems to realize fully the digital ledger paradigm supported by blockchain, and we believe that it is expected to gain popularity in preserving research integrity in the biomedical domain.

Medicines supply chain can potentially benefit from blockchain technology to store drug transaction data to immutably trace and track products from the producer to the consumer and thus combat counterfeit drugs [22,55]. Another important issue is the storage conditions along the supply chain; an interesting application (and the only one with real world demonstration and evaluation) uses ambient temperature sensors to record temperature while drugs are stored and transported and immutably keep such measurements in a public blockchain for transparent inspection [25]. In a similar approach, physical location measurements could be stored in a private manner in a blockchain to allow for readily responding to a medical emergency [38]. Blockchain used as a tamper-proof ledger of the physical location of a material object or of other physical parameters seems a very promising application that follows fully the blockchain ledger paradigm.

Limitations of this scoping review are linked to the literature databases included for publication retrieval. Our search considered some (not all) of the most popular scientific literature indexing systems for information technology and biomedical research. Additionally, search was limited scientific literature, so applications described in grey literature might have been missed. Since the field is still in its infancy, our broad search based only on the term “blockchain” returned a manageable number of records for study. Considering the increasing popularity trends we have seen in this study, future repetitions of a similar scoping review would have to devise smarted search queries to narrow the retrieved records to blockchain applications in the biomedical domain.

## 5 Conclusions

In this paper we present the results of a scoping literature review into the current state of research in the application of blockchain technology in the biomedical domain. Our findings show that the field is still in its infancy. Research maturity of the papers included in this scoping review suggests that blockchain applications in the biomedical domain is still an emerging field. Yearly distribution of related publications supports this finding, showing that research activity in the field starts only in 2016 and is doubled during the first 8 months of 2018, increasing at much higher rates than general position and concept papers in the same field.

In this first 3 years, research is greatly focused on integration, integrity and access control of health records and related patient data. However, other diverse and interesting applications are emerging, addressing medical research, clinical trials, medicines supply chain, and medical insurance. As yet, blockchain has still to find its proper application paradigms, moving away from approaches that discuss storing actual health data chunks in the blockchain, to solutions that use the blockchain as a ledger storing mainly references to data or data hashes.

Apart from identifying new, promising application areas, research should focus on real world evaluation based on large scale deployments that would highlight technology limitations and most likely indicate most suitable applications. Also, special attention should be drawn to privacy, which is not preserved by default in the common blockchain. One issue that is not fully addressed in current literature is the type of blockchain used, namely public, private or consortium. Only a few publications mention the specific type of blockchain used; even then, a proper justification is lacking. We believe that each different blockchain type has its own niche in biomedical applications and we expect that consortium blockchain (with carefully selected miner node owners) might prove the best solution to both guarantee high enough immutability and ensure required efficiency at a tolerable cost.

It is evident that the field still has to find its own forum in the scientific publication realm. So far published literature is greatly scattered in different journals and conferences; the field could benefit from special issues in established scientific journals and dedicated workshops in biomedical conferences, and regular repetitions of similar scoping reviews.

To conclude, this study can become a starting point for future research, demonstration and evaluation of blockchain applications in the biomedical domain, and also a guide for regular, systematic reviews of related research progress.

## Acknowledgment

This work was partly supported by the European Commission FP7-ICT project CARRE (Grant No. 611140) and the corresponding Greek National Matching funds of the General Secretariat of Research and Technology (GSRT). The funders had no role in study design, data collection and analysis, decision to publish, or preparation of the manuscript.
